# Predictors and prognostic implications of hospital-acquired pneumonia in patients admitted for acute heart failure

**DOI:** 10.3389/fcvm.2023.1254306

**Published:** 2023-09-15

**Authors:** Marija Polovina, Milenko Tomić, Mihajlo Viduljević, Nataša Zlatić, Andrea Stojićević, Danka Civrić, Aleksandra Milošević, Gordana Krljanac, Ratko Lasica, Milika Ašanin

**Affiliations:** ^1^ Department of Cardiology, University Clinical Centre of Serbia, Belgrade, Serbia; ^2^ Faculty of Medicine, University of Belgrade, Belgrade, Serbia

**Keywords:** acute heart failure, hospital acquired pneumonia, intensive care unit, mortality, heart failure treatment

## Abstract

**Introduction:**

Data on predictors and prognosis of hospital acquired pneumonia (HAP) in patients admitted for acute heart failure (AHF) to intensive care units (ICU) are scarce. Better knowledge of these factors may inform management strategies. This study aimed to assess the incidence and predictors of HAP and its impact on management and outcomes in patients hospitalised for AHF in the ICU.

**Methods:**

this was a retrospective single-centre observational study. Patient-level and outcome data were collected from an anonymized registry-based dataset. Primary outcome was in-hospital all-cause mortality and secondary outcomes included length of stay (LOS), requirement for inotropic/ventilatory support, and prescription patterns of heart failure (HF) drug classes at discharge.

**Results:**

Of 638 patients with AHF (mean age, 71.6 ± 12.7 years, 61.9% male), HAP occurred in 137 (21.5%). In multivariable analysis, HAP was predicted by *de novo* AHF, higher NT proB-type natriuretic peptide levels, pleural effusion on chest x-ray, mitral regurgitation, and a history of stroke, diabetes, and chronic kidney disease. Patients with HAP had a longer LOS, and a greater likelihood of requiring inotropes (adjusted odds ratio, OR, 2.31, 95% confidence interval, CI, 2.16–2.81; *p* < 0.001) or ventilatory support (adjusted OR 2.11, 95%CI, 1.76–2.79, *p* < 0.001). After adjusting for age, sex and clinical covariates, all-cause in-hospital mortality was significantly higher in patients with HAP (hazard ratio, 2.10; 95%CI, 1.71–2.84; *p* < 0.001). Patients recovering from HAP were less likely to receive HF medications at discharge.

**Discussion:**

HAP is frequent in AHF patients in the ICU setting and more prevalent in individuals with *de novo* AHF, mitral regurgitation, higher burden of comorbidities, and more severe congestion. HAP confers a greater risk of complications and in-hospital mortality, and a lower likelihood of receiving evidence-based HF medications at discharge.

## 1. Introduction

Acute heart failure (AHF) is one of the leading global causes of hospitalisation (1), responsible for −2.2 million hospital admission per year in Europe alone (2). Patients hospitalised for AHF have in-hospital mortality rates ranging between −2.5% and >50%, depending on the clinical severity, and those discharged alive suffer a long-term increased risk of death (−20%) (3, 4). Patients with AHF admitted to the intensive care units (ICU) typically have more severe congestion and/or haemodynamic instability and represent a vulnerable category, often comprising older, frail, and multi-morbid individuals, at higher risk of complications during the hospital stay (5).

Hospital acquired pneumonia is one of the most frequent and serious complications in patients admitted to the ICU. It is defined as an inflammatory condition of the lung parenchyma caused by infectious agents, not present at least 48 h after admission, and does not include patients intubated at admission (6). It is primarily caused by bacterial pathogens (7), and its occurrence exacerbates the risk of respiratory insufficiency, haemodynamic instability and shock and confers the highest mortality among nosocomial infections (6, 8). The pathogenesis of hospital acquired pneumonia includes the aspiration of oropharyngeal pathogens, and the colonization and invasion of the lower respiratory tract, which typically occurs in the presence of compromised host defence mechanisms (9). Rarely pathogens can be directly introduced into the lower airway or spread through the bloodstream from infected intravenous catheters, leading to infection (9). In patients with AHF, congestion in the lower respiratory tract, interstitial and/or alveolar oedema, and engorgement of lymphatic vessels create an environment permissive to bacterial persistence, and impair immune-mediated defence mechanisms, making patients more susceptible to developing pneumonia. Pneumonia development, in turn, can worsen cardiac function due to an increased cardiac workload caused by factors such as tachycardia, oxygen supply-demand imbalance, reduced systemic vascular resistance, and potentially direct myocardial toxic effects of inflammatory mediators (10).

Earlier studies have reported the incidence of hospital acquired pneumonia ranging between 8% and 21% in patients admitted for AHF and its development was associated with the more severe clinical course, longer length of hospitalisation, and greater risk of in-hospital and one-year mortality (11, 12). However, most of the earlier observations were derived from retrospective analysis not specifically conducted in AHF patients admitted to the ICU. Moreover, research focus has shifted over the past few years to SARS-CoV-2 infection and there is a paucity of contemporary data on non-COVID pneumonia. Better understanding of the incidence, risk factors and clinical course of hospital acquired pneumonia in AHF patients in the ICU setting is necessary to inform future risk stratification and management strategies. Therefore, the aim of the present study is to assess the incidence and predictors of hospital acquired pneumonia and its impact on clinical outcomes and management of patients hospitalised for AHF in the ICU.

## 2. Materials and methods

### 2.1. Study design and inclusion criteria

This was a retrospective single-centre analysis of anonymised hospital registry-based dataset of patients admitted for AHF in the cardiology ICU of the Emergency Department of the University Clinical Centre of Serbia, Belgrade, Serbia, between May 2020 and July 2022. The ICU of the Department of Cardiology in the University Clinical Centre of Serbia is a tertiary level facility, which hospitalises patients with acute/critical cardiovascular disorders. AHF was defined by the presence of symptoms and signs of HF, corroborated by radiology evidence of congestion (Kerly B lines, plueral effusion) and elevated natriuretic peptide levels regardless of left ventricular (LV) ejection fraction (LVEF), in line with the European Society of Cardiology (ESC) guidelines on the management of HF (13). Patients with the first episode of AHF (*de novo* AHF) and those with a previous history of chronic HF (decompensated chronic HF) were included. Only patients with a primary diagnosis of AHF, according to the International Statistical Classification of Diseases 10th revision (ICD-10) code I50.* were included. Exclusion criteria were as follows: (1) acute coronary syndrome defined according to the ESC guidelines (14, 15); (2) other cardiovascular emergencies complicated by AHF (e.g., infective endocarditis, pulmonary embolism, high grade atrioventricular block etc); (3) patients in cardiogenic or septic shock at the time of admission; (4) patients with confirmed SARS-CoV-2 or Influenza virus infection; (5) patients with evidence of lower respiratory tract infection at admission (6) patients intubated at the time of admission or within the first 48 h. The study protocol was approved by the institutional ethics review board and informed consent was exempt on the basis of a retrospective design.

### 2.2. Definition of hospital acquired pneumonia

Hospital acquired pneumonia was defined according to the modified Infectious Diseases Society of America and the American Thoracic Society criteria (7, 9), including radiographic evidence of an inflammatory infiltrate that is new or progressive (on chest x-ray and/or computed tomography), along with at least two of the clinical findings suggestive of infection, namely, new onset of fever (>37.5 C), purulent expectoration, leucocytosis, elevated levels of C-reactive protein (CRP), procalcitonin, fibrinogen and decline in oxygenation. Documentation of ICD-10 codes J15.* and J18.* was also required. Only patients who developed pneumonia at least 48 h after admission and were not intubated at the time of admission were considered.

### 2.3. Patient-level data acquisition

Data on baseline demographic characteristics (age, sex), vital signs and HF status at admission (heart rate, systolic and diastolic blood pressure, and manifestations of congestion) were collected from the hospital registry-based dataset. HF status was evaluated in accordance with the ESC guidelines, including standard transthoracic echocardiographic examination performed during hospitalisation to confirm structural and functional alterations (13). Based on echocardiographic exam (performed in all patients), HF was classified as HF with reduced ejection fraction, HFrEF (LVEF ≤40%), HF with mildly reduced ejection fraction, HFmrEF (LVEF >41%–49%) and HF with preserved ejection fraction, HFpEF; the latter two categories were pooled together (HFmrEF/HFpEF) (13). The diagnosis of HFpEF was based the ESC guidelines criteria (13), as follows: (1) presence of symptoms and signs of HF (all patients were admitted with symptomatic acute HF), LVEF ≥50% and “ evidence of cardiac structural and/or functional abnormalities consistent with the presence of LV diastolic dysfunction/raised LV filling pressures, including raised natriuretic peptides”. Diastolic dysfunction was diagnosed in the presence of at least 2 of the 4 criteria (16): (1) left atrial volume index >34 ml/m^2^ (or >40 ml/m^2^ in patients with atrial fibrillation); (2) tricuspid regurgitation jet velocity >2.8 m/s; (3) tissue doppler imaging septal e’ < 7 or lateral e’ < 10; and 4. E/e’ > 14. In patients with only one echocardiographic criterion (due to difficulties imposed by performing exam in severely decompensated patients), HFpEF was considered present if LVEF was ≥50% and elevated admission levels of natriuretic peptides (i.e., NT-fragment of pro-B-type natriuretic peptide, NT-proBNP ≥300 pg/ml) were documented (13). Cardiovascular comorbidities, including a history of arterial hypertension (previous diagnosis of hypertension, including treatment with antihypertensive drugs), ischaemic heart disease (previous diagnosis of angina pectoris, prior myocardial infarction, and/or coronary revascularisation with percutaneous coronary intervention and/or cardiac bypass surgery), dilated cardiomyopathy (previous diagnosis of non-ischaemic dilated LV and systolic dysfunction—LVEF <45%), valvular heart disease (aortic stenosis and/or moderate/severe mitral regurgitation documented by echocardiography), atrial fibrillation (diagnosis of paroxysmal, persistent or permanent atrial fibrillation), peripheral arterial disease (previously confirmed by vascular ultrasound exam or angiography), stroke/transient ischaemic attack (as per medical documentation) were collected. Non-cardiovascular comorbidities including type 2 diabetes mellitus (T2DM—previous diagnosis of T2DM, treatment with glucose-lowering medications or newly diagnosed T2DM during hospitalisation), chronic obstructive pulmonary disease (COPD—previous diagnosis of COPD including prescribed treatment with inhaled bronchodilators/steroids/combined inhalers and/or aminophylline/theophylline), chronic kidney disease (CKD—persistent decrease in estimated glomerular filtration rate, eGFR, <60 ml/min/1.73 m^2^ by Cockcroft–Gault equation) and anaemia (haemoglobin <130 g/L in men, and <120 g/L in women) were assessed from the registry-based dataset. A history of hospital admissions for any cause within the 6 months prior to current hospitalisation was collected. New York Heart Association (NYHA) functional class was defined at admission. Routine laboratory analysis along with inflammatory mediators (maximum white blood cell count, CRP, procalcitonin and fibrinogen levels) and cardiac biomarkers (admission NT-proBNP, and high-sensitivity troponin T levels, hsTnT) were collected. Assessment of congestion included clinical evaluation (dyspnoea, pulmonary rales, jugular venous congestion, lower extremity oedema), radiographic signs at admission chest x-ray (Kerley B lines, unilateral/bilateral pleural effusion), and biomarkers (elevated NT-proBNP levels). Data on the use of inotropes/vasopressors (dopamine, dobutamine, noradrenalin), non-invasive and invasive ventilatory support after the development of pneumonia, and fundamental HF medications prescribed at discharge (angiotensin converting enzyme inhibitors, ACEI; angiotensin-1 receptor blockers, ARB; angiotensin-1 receptor neprilysin inhibitor—sacubitril/valsartan, ARNI; beta-blockers; mineralocorticoid receptor antagonists, MRA; and sodium-glucose type 2 inhibitors, SGLT2I) in surviving patients were documented.

### 2.4. Study outcomes

The primary study outcome was in-hospital all-cause mortality. Secondary outcomes included: (i) length of hospital stay, (ii) requirement for inotropic support and/or non-invasive/invasive ventilatory support and (iii) prescription patterns of fundamental HF drug classes at discharge, depending on the presence of hospital acquired pneumonia, in patients with HFrEF and HFmrEF/HFpEF.

### 2.5. Statistical analysis

Expecting a hazard ratio (HR) of 2.2 for the association between hospital acquired pneumonia and all-cause mortality based on previously published data (11), we calculated a minimum sample size of 514 patients, with a power (1-*β*) of 0.8, and a 2-sided probability of type I error (*α*) of 0.05 (17). Numerical continuous variables are presented as mean and standard deviation or median and interquartile range (IQR), and categorical variables as absolute numbers and percentages. Difference between variables were compared using the parametric Student *t*-test, or non-parametric Man–Whitney *U*-test for numerical variables, and Pearson Chi-square test or Fisher exact probability test for categorical variables, as appropriate. Clinical predictors of the development of hospital acquired pneumonia were analysed in a multivariable logistic regression model, in which clinical variables from Table 1, achieving *p*-value < 0.05 in univariable logistic regression analysis were entered. In cases of a correlation between predictor variables (e.g., pulmonary rales and dyspnoea, *de novo* AHF and history of chronic HF, anaemia and haemoglobin, CKD, serum creatinine and eGFR), the variable with a stronger association in the univariable analysis was used in the multivariable model. Inflammatory mediators were not entered as they depicted maximum values during hospitalisation (including those observed in patients with pneumonia). Independent predictors were defined as variables with a persistent significant association with the development of pneumonia (*p*-value < 0.05) in the multivariable analysis. Cumulative survival rate during hospitalisation in patients with and without pneumonia was assessed with the Kaplan-Meier analysis and compared using the log-rank test. The association between hospital acquired pneumonia and all-cause mortality was analysed in a Cox proportional hazard model adjusted for clinically relevant covariates, including age, sex, NT-proBNP, *de novo* HF, baseline LVEF (continuous) and other variables listed in Table 1, with a *p*-value < 0.05 for the association with all-cause mortality in univariable analyses. If a significant correlation between explanatory variables was identified (e.g., *de novo* HF and a history of HF, systolic and diastolic blood pressure, eGFR and CKD, HFrEF vs. HFmrEF/HFpEF, etc), a variable with a stronger association in univariable analysis was used for adjustment. Time-to-event or time-to-the end of hospitalisation was used to calculate hazard ratios (HR) with the accompanying 95% confidence intervals (CI). The likelihood of the requirement for inotropic or ventilatory support was analysed in a multivariable logistic regression model, which included hospital acquired pneumonia as predictor variable and other clinical variables (with a *p*-value < 0.05 in univariable logistic regression analysis) were used for adjustment. The likelihood or prescribing HF medications was analysed separately in patients with HFrEF and HFmrEF/HFpEF discharged alive, according to the same principle as described above. All analyses were performed using the IBM SPSS software version 29, and 2-sided *p*-value < 0.05 was considered statistically significant.

**Table 1:** Baseline clinical characteristics of the study population.

Variable	All patients, *n* = 638	Without pneumonia, *n* = 501	With pneumonia, *n* = 137	*p*-value
Age (years)	71.6 ± 12.7	71.0 ± 12.5	72.5 ± 14.8	0.067
Sex (male)	395 (61.9)	309 (61.7)	86 (62.7)	0.938
Heart rate (beats per min)	93.9 ± 26.1	94.1 ± 25.7	92.5 ± 27.3	0.806
Systolic blood pressure (mmHg)	118.6 ± 39.9	119.5 ± 39.0	115.7 ± 42.0	0.295
Diastolic blood pressure (mmHg)	73.5 ± 26.1	74.7 ± 18.9	69.9 ± 19.0	0.846
Dyspnoea, *n* (%)	549 (86.0)	416 (83.0)	133 (97.1)	<0.001
Lower extremity oedema, *n* (%)	364 (57.1)	286 (57.0)	78 (56.9)	0.490
Jugular vein distention, *n* (%)	173 (27.1)	135 (26.9)	38 (27.7)	0.832
Pulmonary rales, *n* (%)	462 (72.4)	339 (67.7)	123 (89.8)	<0.001
Kerley B lines, *n* (%)	555 (86.9)	420 (83.8)	135 (98.5)	<0.001
Pleural effusion, *n* (%)	461 (72.2)	344 (68.6)	117 (85.4)	<0.001
De novo AHF, *n* (%)	278 (43.8)	210 (41.9)	68 (49.6)	0.010
Decompensated chronic HF, *n* (%)	360 (56.4)	291 (58.1)	69 (50.4)	0.030
LVEF (%)	34.2 ± 15.7	33.4 ± 15.3	37.0 ± 17.0	0.050
HFrEF, *n* (%)	401 (62.8)	324 (64.7)	77 (56.2)	0.072
HFmrEF/HFpEF, *n* (%)	237 (37.1)	177 (35.3)	60 (43.8)	0.092
NYHA class III, *n* (%)^a^	346 (54.2)	275 (54.8)	71 (51.8)	0.075
NYHA class IV, *n* (%)^a^	292 (45.6)	226 (45.1)	66 (48.1)	0.061
A history of prior hospitalization, *n* (%)	154 (24.1)	113 (22.5)	41 (29.9)	0.022
Cardiovascular comorbidities				
Hypertension, *n* (%)	526 (82.4)	409 (81.6)	117 (85.4)	0.529
Coronary artery disease, *n* (%)	247 (38.7)	199 (39.7)	48 (35.0)	0.340
Dilated cardiomyopathy, *n* (%)	140 (21.9)	113 (22.5)	27 (19.7)	0.564
Aortic stenosis, *n* (%)	82 (12.8)	60 (11.9)	12 (8.7)	0.172
Mitral regurgitation, *n* (%)	198 (31.0)	131 (26.1)	68 (48.9)	<0.001
Atrial fibrillation, *n* (%)	374 (58.6)	290 (57.9)	84 (61.3)	0.525
Peripheral arterial disease, *n* (%)	113 (17.7)	84 (16.8)	29 (21.2)	0.225
Stroke/transient ischaemic attack, *n* (%)	91 (14.3)	58 (11.5)	33 (24.1)	<0.001
Non-cardiovascular comorbidities				
Type 2 diabetes, *n* (%)	252 (35.5)	180 (35.9)	72 (52.5)	<0.001
Chronic kidney disease, *n* (%)	181 (28.3)	120 (23.9)	61 (44.5)	<0.001
Chronic obstructive pulmonary disease, *n* (%)	83 (13.0)	52 (10.4)	31 (22.6)	<0.001
Anaemia, *n* (%)	303 (47.6)	220 (43.9)	83 (60.5)	<0.001
Current smokers, *n* (%)	125 (19.6)	97 (19.3)	28 (20.4)	0.966
Laboratory analysis				
White blood cell count (10^3^/L)	10.6 ± 2.3	8.7 ± 2.4	14.6 ± 3.8	<0.001
CRP (mg/dl)	22.1 ± 13.2	8.9 ± 7.8	33.2 ± 12.3	<0.001
Procalcitonin (ng/ml)	0.6 (0.4–2.7)	0.1 (0.0–0.4)	1.8 (0.0–6.9)	<0.001
Fibrinogen (g/L)	2.3 (1.2–3.2)	2.0 (1.4–3.3)	4.6 (2.4–7.7)	<0.001
Na^+^ (mmol/L)	138.6 ± 5.6	138.5 ± 5.5	139.0 ± 6.0	0.935
Creatinine (μmol/L)	98.1 ± 21.3	92.6 ± 17.5	101.2 ± 23.4	<0.001
Urea (mmol/L)	5.6 ± 2.8	4.9 ± 2.9	6.2 ± 3.4	0.089
eGFR (ml/min/1.73 m^2^)	43.3 ± 21.1	51.4 ± 12.0	37.6 ± 18.1	<0.001
Uric acid (mmol/L)	476.4 ± 74.5	484.3 ± 81.2	472.4 ± 63.8	0.548
Haemoglobin (g/L)	125.1 ± 23.5	133.4 ± 13.8	116.5 ± 12.6	<0.001
NT-proBNP (pg/ml)	5293 (2795–11328)	4865 (998–7853)	7414 (1189–12345)	<0.001
hs-TnT (ng/ml)	78.0 (41.0–154.4)	68.4 (31.2–146.9)	78.3 (44.2–166.7)	0.089

CRP, C reactive protein; eGFR, estimated glomerular filtration rate; HFmrEF, heart failure with mildly reduced ejection fraction; HFpEF, heart failure with preserved ejection fraction; HFrEF, heart failure with reduced ejection fraction; hsTnT, high sensitivity troponin T; LVEF, left ventricular ejection fraction; NT-proBNP, N-terminal pro B type natriuretic peptide; Na+, serum sodium level; NYHA, New York Heart Association.

^a^ There were no asymptomatic or mildly symptomatic patients (i.e., NYHA functional class I or II).

## 3. Results

### 3.1. Baseline patient characteristics and the incidence of hospital acquired pneumonia

The study included 638 patients admitted to the cardiology ICU for AHF (mean age, 71.6 ± 12.7 years, male 61.9%). Hospital acquired pneumonia was documented in 137 patients (21.5%) after a median of 5.5 days from admission (IQR, 3.0–7.8 days). Microbiological confirmation was available in a subset of patients who subsequently required intubation and mechanical ventilation, in whom positive endotracheal aspirate revealed the following pathogens: Acinetobacter spp, Klebsiella pneumonia, Pseudomonas aeruginosa or Staphylococcus aureus. Baseline clinical characteristics of the entire cohort and according to the presence of pneumonia are presented in Table 1.

Pneumonia occurred more frequently in patients with *de novo* AHF compared with those with decompensated chronic HF (49.6% vs. 41.9%, *p* = 0.010), Table 1. Patients who developed pneumonia had more severe evidence of congestion on admission in terms of dyspnoea (97.1% vs. 83.0%), pulmonary rales (89.8%. vs. 67.7%) and radiographically documented Kerley B lines (98.5% vs. 83.8%) and pleural effusions (85.4% vs. 68.6%), all *p*-values < 0.001, Table 1. Patients with hospital acquired pneumonia more frequently had a history of a previous hospitalisation withing the past 6 months (29.5% vs. 25.5%%, *p* = 0.022). Mitral regurgitation and a history of stroke/transient ischaemic attack were more prevalent in patients with pneumonia compared to those without pneumonia (mitral regurgitation, 48.9% vs. 26.1%; prior stroke, 24.1% vs. 11.5%, respectively both *p*-values < 0.001), while there was no difference in other cardiovascular comorbidities, Table 1. Mean LVEF was slightly higher in patients with pneumonia compared to the rest of the cohort (*p* = 0.050). Non-cardiovascular comorbidities, including T2DM (52.5% vs. 35.9%, *p*-value < 0.001), COPD (22.6% vs. 10.4%, *p*-value < 0.001), CKD (44.5% vs. 23.9%, *p*-value < 0.001) and anaemia (60.5% vs. 43.9%, *p*-value < 0.001) were significantly more frequent in patients with pneumonia. There was no difference in the smoking status. Patients with pneumonia had higher maximum levels of inflammatory biomarkers, and higher admission levels of serum creatinine and NT-proBNP, Table 1. Admission eGFR and haemoglobin levels were lower in patients with pneumonia, and there were no significant differences in other laboratory values, including hsTnT, Table 1.

### 3.2. Predictors of the development of hospital acquired pneumonia

Variables significantly associated with the development of hospital acquired pneumonia are presented in Table 2. In multivariable analysis, the development of pneumonia was independently predicted by radiographic evidence of pleural effusion at admission, *de novo* AHF, presence of mitral regurgitation, a history of stroke/transient ischaemic attack, T2DM and CKD, and increased admission levels of NT-proBNP (≥ the median value of 5,293 pg/ml), Table 2.

**Table 2:** Univariable and multivariable predictors of the development of hospital acquired pneumonia.

Variable	Univariable analysis^a^ OR (95% CI)	*p*-value	Multivariable analysis OR (95% CI)	*p*-value
Dyspnoea	1.31 (1.11–2.11)	0.007	1.20 (0.97–1.67)	0.346
Kerley B lines	1.23 (1.09–1.98)	0.001	1.19 (0.96–1.78)	0.346
Pleural effusion	2.51 (1.50–4.18)	<0.001	2.70 (1.49–4.06)	<0.001
De novo AHF	1.65 (1.12–2.41)	0.010	1.85 (1.14–3.08)	0.003
A history of prior hospitalisation	1.24 (1.13–2.41)	0.031	1.10 (0.90–1.96)	0.189
LVEF (continuous)	1.01 (1.00–1.03)	0.028	1.01 (0.89–1.04)	0.055
Mitral regurgitation	3.22 (2.81–3.84)	<0.001	2.52 (2.19–2.96)	<0.001
Stroke/transient ischaemic attack	2.28 (1.76–3.15)	<0.001	1.20 (1.07–2.13)	0.020
Type 2 diabetes	1.64 (1.24–1.95)	0.018	1.61 (1.30–2.14)	0.009
Chronic kidney disease	1.67 (1.27–2.17)	0.009	1.24 (1.12–1.79)	0.005
Chronic obstructive pulmonary disease	1.28 (1.17–2.20)	0.035	1.07 (0.87–1.82)	0.118
Anaemia	1.24 (1.04–1.83)	0.041	1.21 (0.84–1.33)	0.228
NT-proBNP (≥5293 pg/ml)^b^	3.31 (2.71–5.21)	<0.001	3.25 (3.16–4.05)	<0.001

CI, confidence interval; NT-proBNP, N-terminal pro B type natriuretic peptide; OR, Odds ratio.

^a^ Only variables with a significant association with the development of pneumonia in univariable analysis are presented.

^b^ median value of NT-prBNP in the study population.

### 3.3. Association between hospital acquired pneumonia and clinical outcomes

**Figure 1:**
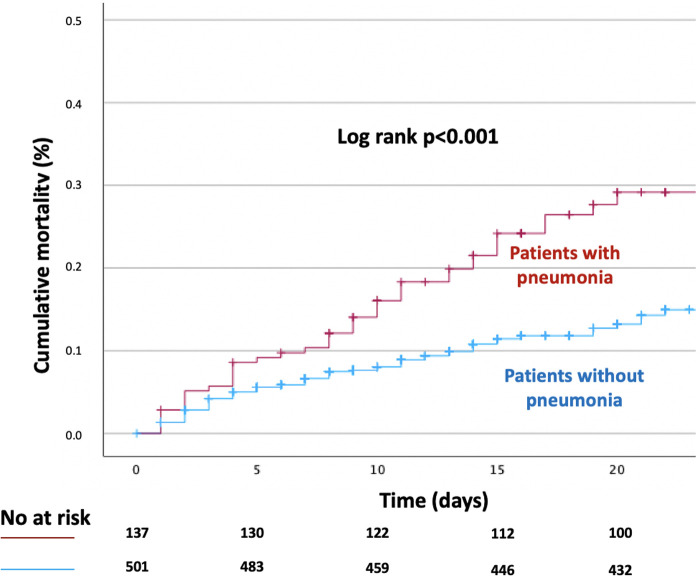
Kaplan-Meier time-to-event curve for in-hospital all-cause death according to the presence of hospital acquired pneumonia.

A total of 106 (16.6%) patients died during hospitalisation. In-hospital all-cause mortality rates were significantly higher among patients with hospital acquired pneumonia (27.0%) compared with patients without pneumonia (13.8%), *p*-value < 0.001. Cumulative Kaplan-Meier time-to-event curves in patients with and without pneumonia are presented in Figure 1. After adjusting for age, sex and other clinically relevant covariates, the development of pneumonia was independently associated with a significantly higher risk of in-hospital all-cause mortality (adjusted HR, 2.10; 95% CI, 1.71–2.84; *p*-value < 0.001).

**Figure 2:**
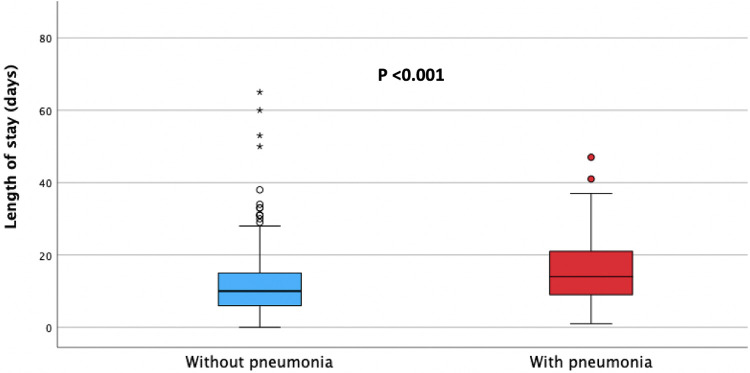
Median length of hospital stay in patients with (red) and without (blue) hospital acquired pneumonia.

The median length of hospitalisation was significantly longer in patients with hospital-acquired pneumonia (median, 14.5 days, IQR 9.5–22 days) compared to patients without pneumonia (median, 10 days, IQR 6–16 days), *p* < 0.001, as presented in Figure 2.

During hospitalisation, inotropes/vasopressors and ventilatory support were required in 37.7% and 26.0% of the total study population; patients with hospital acquired pneumonia were significantly more likely to require either type of the support (inotropes/vasopressors, adjusted odds ratio, OR, 2.31, 95% CI, 2.16–2.81, *p*-value <0.001; ventilatory support, adjusted OR, 2.11, 95% CI, 1.76–2.79, *p*-value < 0.001), Table 3.

**Table 3:** Association between hospital acquired pneumonia and treatment during hospitalisation.

Variable	All patients, *n* (%)	Without pneumonia, *n* (%)	With pneumonia, *n* (%)	Unadjusted OR (95% CI)	*p*-value	Adjusted OR (95% CI)	*p*-value
Inotropes/vasopressors	241 (37.7)	170 (33.9)	71 (51.8)	2.49 (1.69–3.70)	<0.001	2.31 (2.16–2.89)	<0.001
Ventilatory support	166 (26.0)	116 (23.1)	50 (36.5)	2.37 (1.28–3.99)	<0.001	2.11 (1.76–2.78)	<0.001

ACEI, angiotensin converting enzyme inhibitors; ARB, angiotensin-1 receptor blocker; ARNI, angiotenin-1 receptor neprilysin inhibitor; HFmrEF, heart failure with mildly reduced ejection fraction; HFpEF, heart failure with preserved ejection fraction; HFrEF, heart failure with reduced ejection fraction; MRA, mineralocorticoid receptor antagonist; SGLT2, sodium glucose cotransporter type 2.

Prescription patterns of key evidence-based HF medications in patients discharged alive with HFrEF and HFmrEF/HFpEF are presented in Tables 4, 5, respectively. A total of 77.2% and 58.3% of HFrEF patients were prescribed at discharge with renin angiotensin system inhibitors (ACEI/ARB/ARNI) and beta-blockers, respectively, Table 4. Patients who had hospital acquired pneumonia were less likely to receive either of those drug classes compared to patients without pneumonia (ACEI/ARB/ARNI, 43.4% vs. 84.2%, adjusted OR 0.75, 95% CI, 0.51–0.96, *p* = 0.018; beta blockers, 41.5% vs. 61.7%, adjusted OR 0.56, 95% CI, 0.34–0.90, *p* = 0.008, respectively), Table 4. MRA and SGLT2I were prescribed in 50.1% and 44.3% of HFrEF patients, and there was no significant difference in the prescription rates between patients with and without pneumonia, Table 4.

**Table 4:** Association between hospital acquired pneumonia and heart failure medications prescribed at discharge in patients with heart failure and reduced ejection fraction.

Heart failure medications	All patients, 317 (79)	Without pneumonia, 264 (81.5)^a^	With pneumonia, 53 (68.8)^a^	Unadjusted OR (95% CI)	*p*-value	Adjusted OR (95% CI)	*p*-value
ACEI/ARB/ARNI	245 (77.2)	222 (84.2)	23 (43.4)	0.76 (0.47–0.97)	0.022	0.75 (0.51–0.96)	0.018
Beta-blockers	185 (58.3)	163 (61.7)	22 (41.5)	0.51 (0.30–0.87)	0.013	0.56 (0.34–0.90)	0.008
MRA	159 (50.1)	136 (51.5)	23 (43.4)	0.78 (0.45–1.30)	0.331	0.81 (0.46–1.10)	0.427
SGLT2 inhibitor	140 (44.3)	116 (43.9)	24 (45.2)	1.08 (0.67–1.18)	0.728	0.98 (0.72–1.12)	0.744

ACEI, angiotensin converting enzyme inhibitors; ARB, angiotensin-1 receptor blocker; ARNI, angiotenin-1 receptor neprilysin inhibitor; HFmrEF, heart failure with mildly reduced ejection fraction; HFpEF, heart failure with preserved ejection fraction; HFrEF, heart failure with reduced ejection fraction; MRA, mineralocorticoid receptor antagonist; SGLT2, sodium glucose cotransporter type 2.

^a^ % of patients discharged alive in relation to all patients with HFrEF, and those with and without pneumonia.

**Table 5:** Association between hospital acquired pneumonia and heart failure medications prescribed at discharge in patients with heart failure and mildly reduced/preserved ejection fraction.

Heart failure medications	Patients, 215 (90.7)^a^	Without pneumonia, 168 (94.9)^a^	With pneumonia, 53 (68.8)^a^	Unadjusted OR (95% CI)	*p*-value	Adjusted OR (95% CI)	*p*-value
ACEI/ARB/ARNI	174 (80.9)	134 (79.7)	36 (76.5)	0.97 (0.81–1.23)	0.854	0.99 (0.85–1.16)	0.911
Beta-blockers	97 (45.1)	83 (49.4)	14 (29.8)	0.52 (0.31–0.78)	<0.001	0.51 (0.38–0.73)	<0.001
MRA	93 (43.2)	77 (45.8)	16 (34.0)	0.78 (0.56–0.87)	<0.001	0.75 (0.52–0.84)	0.010
SGLT2 inhibitor	56 (26.0)	42 (25.0)	14 (29.8)	1.13 (0.87–1.44)	0.589	1.09 (0.89–1.28)	0.346

ACEI, angiotensin converting enzyme inhibitors; ARB, angiotensin-1 receptor blocker; ARNI, angiotenin-1 receptor neprilysin inhibitor; HFmrEF, heart failure with mildly reduced ejection fraction; HFpEF, heart failure with preserved ejection fraction; HFrEF, heart failure with reduced ejection fraction; MRA, mineralocorticoid receptor antagonist; SGLT2, sodium glucose cotransporter type 2.

^a^ % of patients discharged alive in relation to all patients with HFmrEF/HFpEF, and those with and without pneumonia.

Among patients with HFmrEF/HFpEF, ACEI/ARB/ARNI, beta-blockers, MRA and SGLT2I were prescribed in 80.9%, 45.1%, 43.2%, and 26.0% of patients, respectively, Table 5. Patients with hospital acquired pneumonia had a lower likelihood of being prescribed beta-blockers and MRA (beta-blockers, 29.8% vs. 49.4%, adjusted OR, 0.51, 95% CI, 0.38–0.73, *p* < 0.001; MRA, 34.0% vs. 45.8%, adjusted OR 0.75, 95% CI, 0.52–0.84; *p* = 0.010, respectively), whilst there was no difference in the prescription of ACEI/ARB/ARNI and SGLT2I, Table 5.

## 4. Discussion

There are three main findings of the present study in a cohort of 638 patients admitted for AHF: (i) hospital acquired pneumonia is a frequent complication of hospitalisation in the ICU, affecting 21.5% of the patients; (ii) its occurrence is more frequent in patients with *de novo* AHF and is predicted by the more severe markers of congestion (i.e., pleural effusions and higher NT-proBNP levels) and presence of mitral regurgitation and non-cardiovascular comorbidities, including a history of stroke/transient ischaemic attack, T2DM and CKD; (iii) the development of pneumonia is associated with a greater requirement for haemodynamic and ventilatory support, longer length of hospitalisation, and a significantly increased in-hospital mortality, whilst the recovered patients have a lower likelihood of receiving evidence-based treatment for HF at discharge.

Previous studies have reported variable incidence of hospital acquired pneumonia in patients admitted for AHF ranging from 8%–21%, and up to 25% in critically ill individuals (11, 12, 18). In the present study, which included only cases of AHF in need of the cardiology ICU management, pneumonia developed in approximately one in five of the admitted patients. Of note, our analysis was restricted to non-intubated patients at the time of admission or within the first 48 h and did not account for the ventilator-associated pneumonia. The median time to the development of pneumonia was 5.5 days, consistent with the greater prevalence of the “late-onset” pneumonia (i.e., pneumonia occurring 5 days or more after admission), which is more likely to be caused by multi-drug resistant pathogens and associated with higher morbidity and mortality (9). This is consistent with culture isolates in the present study revealing gram-negative bacteria and Staphylococcus aureus, usually of the drug-resistant type.

In the present study, pneumonia developed more frequently among patients with the first episode of AHF (*de novo* AHF), compared with decompensated chronic HF, which has not been previously described. A possible explanation of this new observation is that patients with *de novo* AHF, being naïve to the diuretic treatment prior to hospitalisation, might have suffered more pronounced congestion, which had created a host environment more susceptible to nosocomial infection. Clinical course, risk of complications and outcomes of patients with *de novo* AHF as compared with decompensated chronic HF have not been consistent in previous reports (19–21). A meta-analysis of 15 studies (a total of 38,320 patients) has suggested lower mortality but a greater risk of nosocomial infections in *de novo* AHF compared with decompensated chronic HF, which is consistent with our findings (22). Moreover, our study has characterised patients at risk of acquiring pneumonia as individuals with the more pronounced congestion, documented by either radiographic evidence of pleural effusions or significantly elevated natriuretic peptide levels. It is possible that a strategy of more rapid decongestion after hospital admission (i.e., with a combination of diuretics) (23), could have a favourable impact on lowering the risk of infection and improving outcomes in those patients, which deserves future prospective evaluation. Furthermore, early initiation of antibiotic treatment in patients with suspected hospital acquired pneumonia, guided by clinical criteria alone, is strongly recommended to improve prognosis (7). The treatment may be initiated empirically, informed by the local distribution of pathogens and their antibiotic susceptibilities, and then corrected according to culture isolates (7).

The population of the present study was mostly comprised of the elderly individuals with a high prevalence of cardiovascular and non-cardiovascular comorbidities, in line with the characteristics of patients with AHF from several recent multinational registries (3, 21, 24). Similar to earlier reports, we have also observed that the presence of comorbidities increased the risk of acquiring pneumonia (11, 12). In particular, the presence of mitral regurgitation, a history of stroke/transient ischaemic attack, T2DM and CKD have emerged as significant predictors of pneumonia, independently of other clinical characteristics. Recently, mitral regurgitation has been associated with a worse clinical profile of congestion in AHF, which may have been a predisposing factor for pneumonia (25), whilst patients with a history of stroke may have had a higher risk of aspiration due to their residual neurological deficit. T2DM and CKD have been well established predictors of adverse outcomes in AHF (26, 27), however, the present study provides a new observation of their independent association with a higher risk of developing pneumonia.

Our findings confirm earlier observations that hospital acquired pneumonia is associated with significantly impaired short-term outcomes, even in the era of contemporary treatment and advanced life support provided in the cardiology ICU. Earlier studies have suggested that the development of pneumonia increased the length of hospital stay by an average of 7–9 days per patient (9) and in our study, the median length of hospital stay was prolonged by 4.5 days in patients with pneumonia. Furthermore, a significant proportion of patients with pneumonia suffered a haemodynamic (51.8%) and respiratory (36.5%) compromise with the requirement for initiation of inotropic and/or ventilatory support, which may have deteriorated their cardiovascular illness and provoked a downward spiral leading to imminent demise. It is therefore not surprising that the observed in-hospital all-cause mortality rates were doubled in the presence of pneumonia (27.0% vs. 13.8%), and the relative risk of death was over two-fold higher in patients with pneumonia compared to the rest of the cohort, even after adjustment for major clinical covariates. This is in line with previous studies reporting excess mortality in individuals with hospital acquired pneumonia reaching 30%–70% among the critically ill patients (9). A recent Japanese study has reported lower in-hospital mortality (12%) compared to our findings (11), which can be explained by inclusion of patients with the more severe HF in the present study. The Japanese study has also indicated an excess mortality in patients requiring admission to the ICU, as well as a greater risk of worsening HF and impaired long-term survival following nosocomial pneumonia (11). A British study has also demonstrated almost two-fold increased hazard ratios for in-hospital mortality in patients with pneumonia (12), which is consistent with our observations.

The present study has provided a new insight into the adverse impact on hospital acquired pneumonia on prescription patterns of evidence-based therapies for HF in patients discharged alive. In patients with HFrEF, we have observed a significantly lower prescription rates of ACEI/ARB/ARNI and beta-blockers (43.5% and 41.5%, respectively) in individuals recovering from pneumonia, compared to patients without pneumonia (84.2% and 61.7%, respectively). Following adjustment for relevant clinical variables, hospital acquired pneumonia was identified as an independent predictor of a lower likelihood of the prescription of either drug classes (odds ratio for ACEI/ARB/ARNI and betablockers, 0.75 and 0.56, respectively). There was no significant difference in the prescription of MRA and SGLT2I in patients with HFrEF with and without pneumonia. Interestingly, SGLT2I uptake has slightly exceeded that of other drug classes in HFrEF patients recovering from pneumonia, albeit the official recommendation for their use in HFrEF has been issued in the 2021 Guidelines (approximately halfway through the study) (13). It is possible that lower prescription rates of ACEI/ARB/ARNI and beta-blockers reflect a higher incidence of haemodynamic and respiratory insufficiency occurring during hospitalisation in patients with pneumonia, which had led to a greater reluctance among the treating cardiologists to initiate these medications before discharge.

Although the ESC guidelines at the time when the study was conducted have not provided a recommendation for evidence-based therapies for HFmrEF and HFpEF, it has been a longstanding practice to prescribe renin-angiotensin-aldosterone system inhibitors and beta-blockers for the management of comorbidities (e.g., hypertension, atrial fibrillation etc.) in those patients (13, 28). This is supported by recent observational and clinical trial data indicating their broad uptake in patients discharged with HFmrEF/HFpEF (29, 30). In the present study, prescription rates of beta-blockers and MRA at discharge in patients with HFmrEF/HFpEF were lower (29.8% and 34.0%, respectively) compared with patients without pneumonia (49.4% and 45.8%, respectively) and the development of pneumonia was independently associated with a lower likelihood of providing beta-blockers and MRA at discharge in patients with HFmrEF/HFpEF. There was no difference in the prescription of ACEI/ARB/ARNI and SGLT2I. Overall prescription of SGLT2I in patients with HFmrEF/HFpEF was higher than in some of the contemporary studies (29, 30). This may reflect the high prevalence of concomitant T2DM, but also greater confidence among the treating physicians regarding the safety of SGLT2I initiation early after stabilisation in AHF. Of note, lower overall prescription of evidence-based therapies for HF at discharge in patients recovering from pneumonia may be an important contributor to their late adverse prognosis documented in earlier studies of hospital acquired pneumonia in AHF, which deserves further evaluation (11, 12).

### 4.1. Study limitations

Several limitations of the present study need to be acknowledged. This was a retrospective analysis of a single-centre hospital registry-based data with a limited sample size, which imposes a limitation to the generalisability of our findings. Due to the retrospective design, there is a possibility that some cases of infection other than pneumonia may have been misdiagnosed, although the diagnosis of pneumonia was based on major clinical guidelines and confirmed by documentation of specific ICD-10 codes. We did not document the reasons and circumstances leading to HF decompensation and several clinical characteristics were not systematically recorded and could not be used as covariates in the analyses, including several risk factors for pneumonia (e.g., recent hospitalisation prior to current admission, residence in the nursing home, immunosuppressive disease or therapy), pathogens isolated from microbiological samples, antibiotic regimens used, mechanical ventilation modes and duration, and reasons for not prescribing certain HF medications. Pneumonia was microbiologically confirmed in a subset of patients requiring intubation, with a positive endotracheal aspirate revealing typical gram-negative bacteria and Staphylococcus aureus, which does not rule out other causative microorganisms. Also, data on a history of previous pneumonia, or potential antibiotic use prior to hospital admission were not available, albeit this information could have improved our understanding of the risk of developing pneumonia and antimicrobial susceptibility of the causative pathogens. Furthermore, the present study has not assessed the factors contributing to the development of pneumonia or cardiovascular deterioration during pneumonia. On the basis of these limitations, observations made in the present study should be interpreted as hypothesis generating and may stimulate further prospective evaluation.

## 5. Conclusions

Hospital acquired pneumonia is a frequent complication in contemporary patients with AHF admitted to the ICU. Its occurrence is predicted by the more severe markers of congestion (in particular pleural effusions and higher admission lelevs of natriuretic peptides) and is more frequent in patients with *de novo* AHF, particularly in the presence of comorbidities. The development of hospital acquired pneumonia is associated with a longer and more complicated clinical course, including greater risk of haemodynamic and respiratory deterioration. Consequently, hospital acquired pneumonia is an important independent predictor of increased in-hospital all-cause mortality. Finally, patients recovering from pneumonia face a lower likelihood of being discharged with the appropriate medications for HF, which may affect their long-term outcomes. Given the clinical significance of these observations, further prospective research is required into the optimal preventive and management strategies of AHF patients suffering nosocomial pneumonia.

## Data Availability

The raw data supporting the conclusions of this article will be made available by the authors, without undue reservation.
